# Multicentric carpotarsal osteolysis syndrome with variants of MAFB gene: a case report and literature review

**DOI:** 10.1186/s12969-024-00964-6

**Published:** 2024-03-13

**Authors:** Xianfei Gao, Xiang Fang, Danping Huang, Song Zhang, Huasong Zeng

**Affiliations:** 1 Department of Pediatric Allergy, Immunology & Rheumonoly, Guangzhou Women and Children’s Medical Center, Guangzhou Medical University, Guangzhou, 510623 China; 2 Guangdong Province Key Laboratory of Allergy & Clinical Immunology, Guangzhou Medical, Guangzhou, 510623 China; 3 Medical Research Institute, Guangdong Provincial People’s Hospital (Guangdong Academy of Medical Sciences), Southern Medical University, Guangzhou, China

**Keywords:** Multicentric carpotarsal osteolysis, MCTO, MAFB mutation, Denosumab

## Abstract

**Background:**

Multicentric carpotarsal osteolysis (MCTO) is a rare genetic disorder characterized by the progressive loss of bone in the hands, feet, and other skeletal structures. It presents with symptoms that may resemble those of juvenile idiopathic arthritis, making diagnosis challenging for clinicians. The identification of MAF BZIP Transcription Factor B (MAFB) mutations as significant contributors to MCTO represents a major breakthrough in our understanding of the pathogenesis of this rare skeletal disorder.

**Case presentation:**

Our objective was to present the phenotype, treatment, and outcome of a patient with a variant of MAFB-induced MCTO to broaden the range of clinical features associated with MCTO and share our clinical experience for improved diagnosis and treatment. In our case, early MRI examination of the bones and whole exome sequencing enabled an early and accurate MCTO diagnosis, and timely Denosumab administration resulted in no deterioration.

**Conclusion:**

This suggests that MRI examination and whole exome sequencing should be considered when MCTO is suspected, and Denosumab might be an option in the treatment of MCTO.

## Background

Multicentric carpotarsal osteolysis (MCTO) is a genetic skeletal disorder characterized by progressive destruction and resorption of bones in the hands, feet, and other affected joints [1, 2]. Diagnosing MCTO poses significant challenges for healthcare professionals primarily because it shares similarities with various other conditions such as Juvenile Arthritis (JIA) which may lead to misdiagnosis if not carefully evaluated [3–5]. MAFB is a transcription factor that plays an essential role in skeletal development and maintenance. The identified mutations within the coding region of this gene disrupt its normal function, leading to dysregulation of osteoclast activity, cells responsible for bone resorption, resulting in excessive bone loss observed in individuals affected by MCTO [6–8]. More case reports exhibiting clinical manifestation and treatment experience are required to understand the phenotype of mutations within MABF further and develop appropriate treatment options [9–13].

In this article, we present the case of a 4-year-old boy with MCTO induced by mutation of the MABF gene with a literature review to discuss the clinical manifestations, diagnosis, and treatment with MCTO.

## Case presentation

**Fig. 1:**
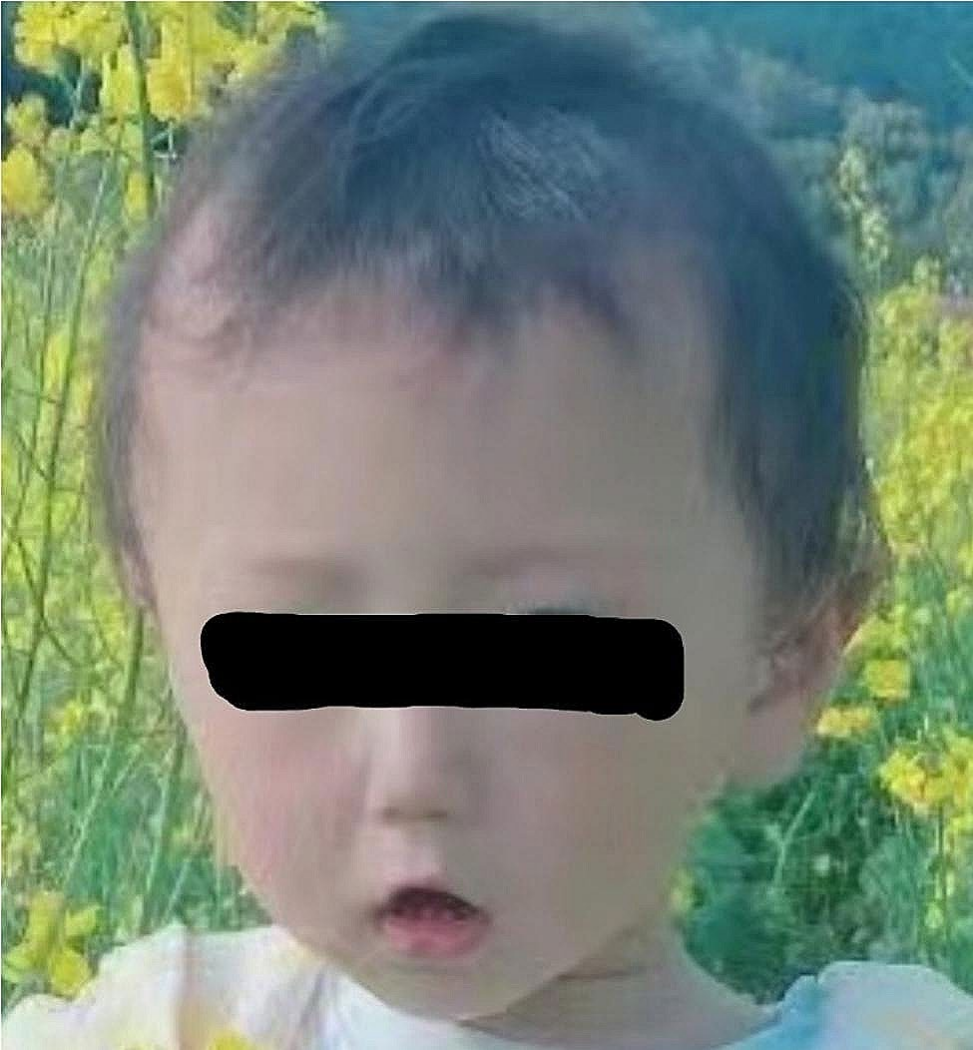
Facial features of the patient showed triangular face, prominent forehead, narrow nose, and micrognathia

**Fig. 2:**
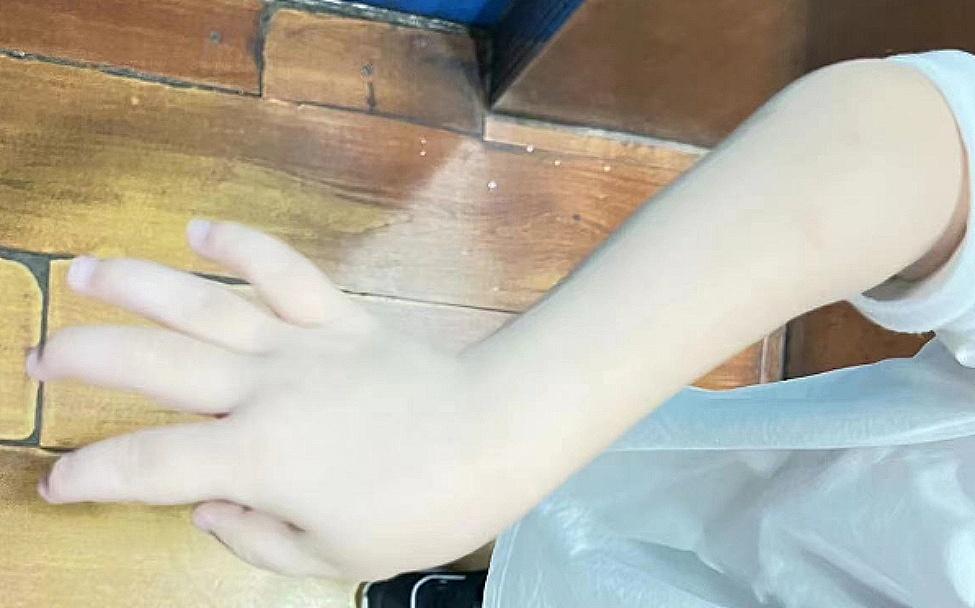
Ulnar deviation

**Fig. 3:**
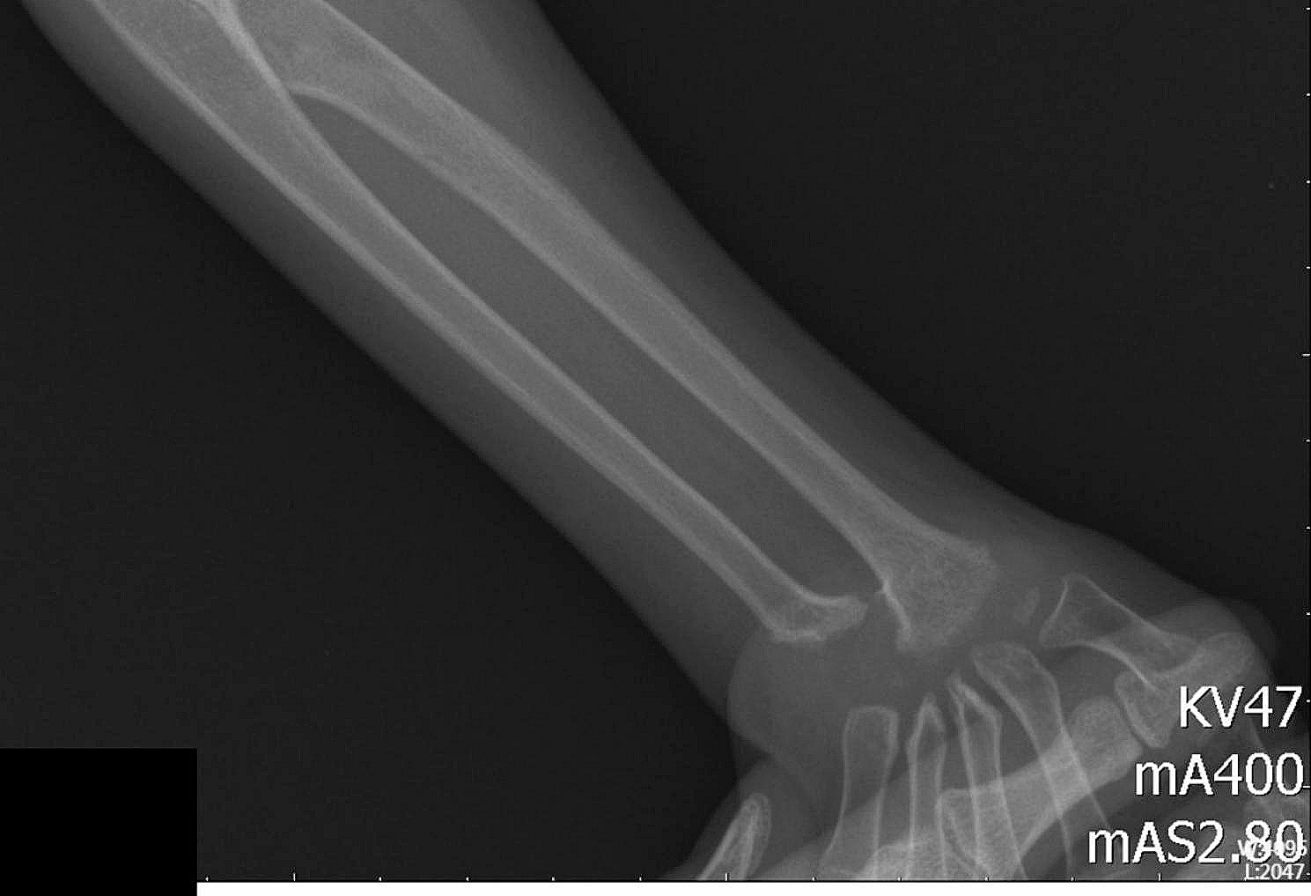
X-ray of right hand of the patient at 3 years of age showed swelling of the soft tissue of elbow joint. Deformities of the proximal ends of the metacarpals and distal ulna and radius were noted

**Fig. 4:**
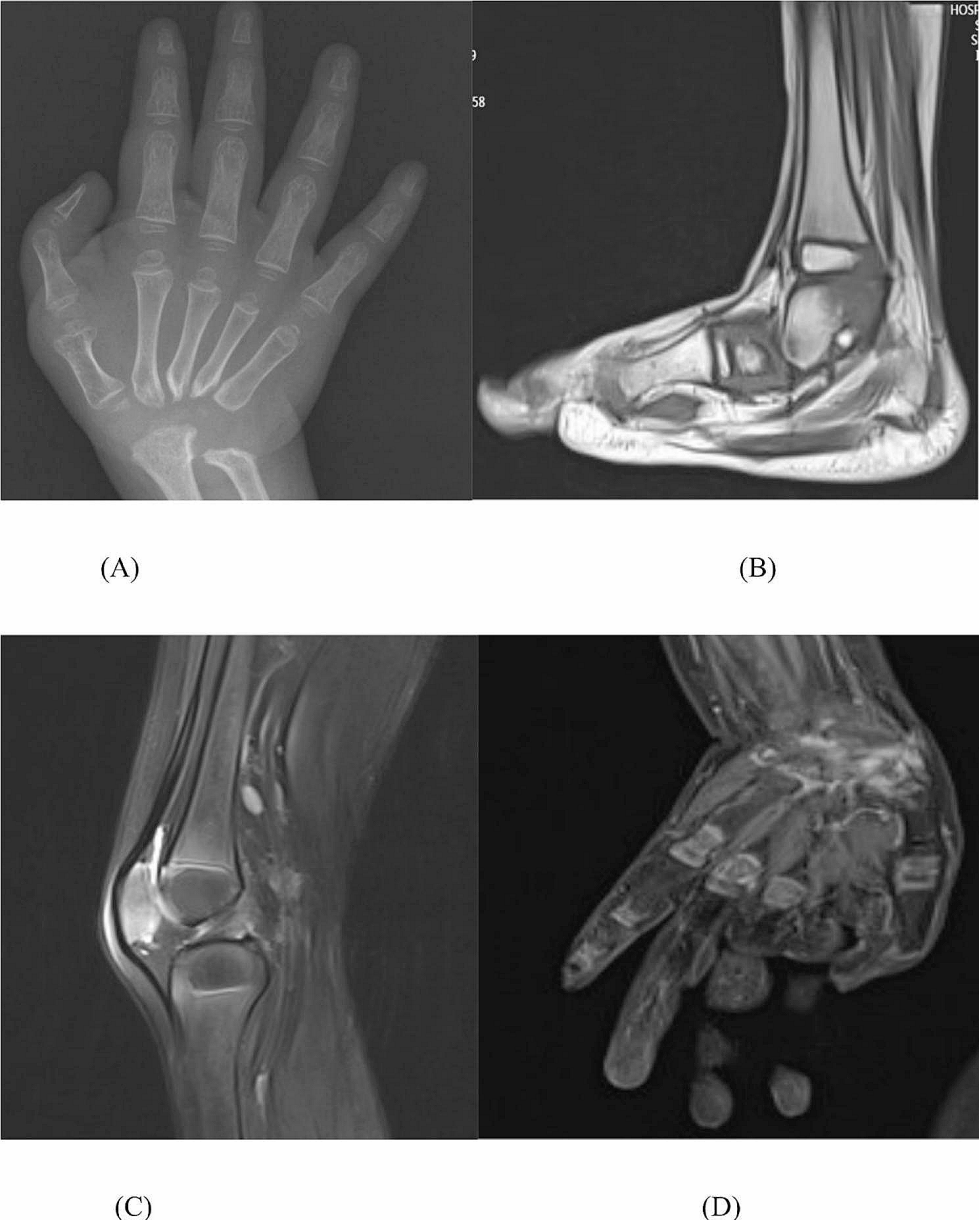
MR of the patient at 3 years of age showed bone destruction of right distal radius, ulna, carpal bone, proximal metacarpal bone (**A**) and left talus, navicular bone, cuneiform bone (**B**) and intra-articular effusions and synovitis of left knee (**C**), and metatarsophalangeal joints and interdigital joints (**D**)

The patient was a 4-year-old Chinese boy, born to healthy non-consanguineous parents. There was no significant familial history. He presented at birth with polydactyly of fingers and underwent surgery without complication at age 1 year. His rheumatologic symptoms began at 2.5 years with pain and swelling in both wrists and hands, ulnar deviation, left ankle, and both metacarpophalangeal joints. His facial features included a triangular face, prominent forehead, narrow nose, and micrognathia (Fig. 1). At the age of 3 years old. He had a pain-avoiding gait in his painful left leg. Swelling and limited extension in his left knee and right elbow, left clubfoot, swelling and stiffness in bilateral metacarpophalangeal joints, and ulnar deviation of both wrists (Fig. 2) had already developed. The right-hand X-ray showed swelling of the soft tissue of the elbow joint. Deformities of the proximal ends of the metacarpals and distal ulna and radius were noted (Fig. 3). MRI showed bone destruction of the right distal radius, ulna, carpal bone, proximal metacarpal bone and left talus, navicular bone, cuneiform bone, and intra-articular effusions and synovitis of the left knee, and metatarsophalangeal joints and interdigital joints (Fig. 4). Osteolysis involving carpal bones, metatarsal bones, metacarpal bones, right radius, and ulna led to the diagnosis of MCTO.

There was no evidence of renal involvement with 24-hour urine protein ( 0.01-0.02 g/24 h ), urine routine ( red cell (0–4/ul), white cell (0–3/ul), protein ( - ) ), BUN (Blood urea nitrogen) (3.81-5.23mmol/L) and creatinine (23-26umol/L). The inflammation values are normal with ESR (Erythrocyte Sedimentation Rate) (10-15 mm/h), CRP (The C-reactive protein) (0.5-4.6 mg/L), TNF-β(Tumor necrosis factor-β) (0.63pg/ml ), IL-12p70 (Interleukin-12p70) (0.90 pg/ml ), IL-1β (0.55 pg/ml), IL-10 (0.65 pg/ml), IL-6 (0.87 pg/ml), TNF-a (0.54 pg/ml), IL-2 (0.68 pg/ml), IFN-r (0.38 pg/ml), IL-17 F (2.78 pg/ml), IL-8 (2.90 pg/ml), IL-4 (0.46 pg/ml), IL-5 (0.52 pg/ml), IL-17 A (0.60 pg/ml). No abnormal eye movements, strabismus, or diplopia were detected on ophthalmological examination. These were detected in the urine during regular monitoring.

**Fig. 5:**
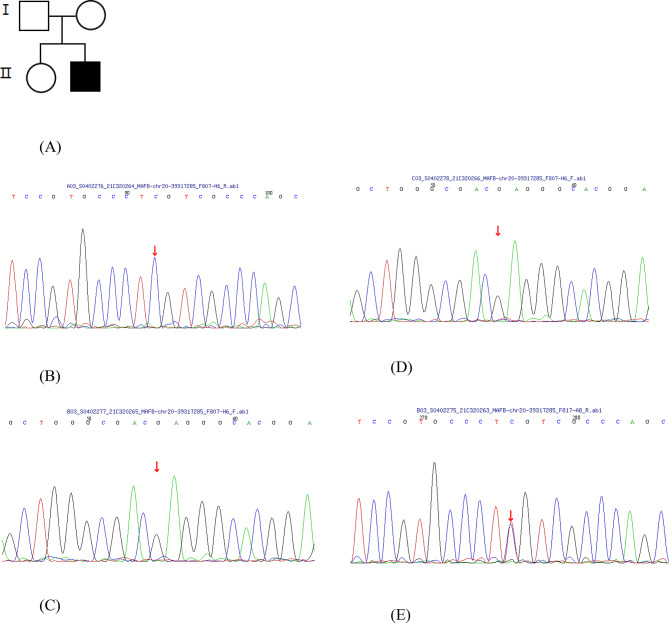
Pedigree and MAFB partial electropherograms. Family pedigree (**A**), DNA sequencing from his father (**B**), his mother (**C**), his healthy sibling (**D**), and the proband (**E**) demonstrating a heterozygous missense mutation at nucleotide 206 from C to T (c.206 C > T) that predicts the change of amino acid at codon 69 from serine to leucine (p.Ser69Leu)

Whole genome sequencing was performed on the patient and showed a missense mutation in the region of MAFB. And this detected a heterogenous missense mutation in the MAFB gene (c.206 C > T) with Thymine replacing Cytosine at nucleotide 206. This change resulted in a change of amino acid number 69 from serine to leucine (p.S69L) (Fig. 5).

**Fig. 6:**
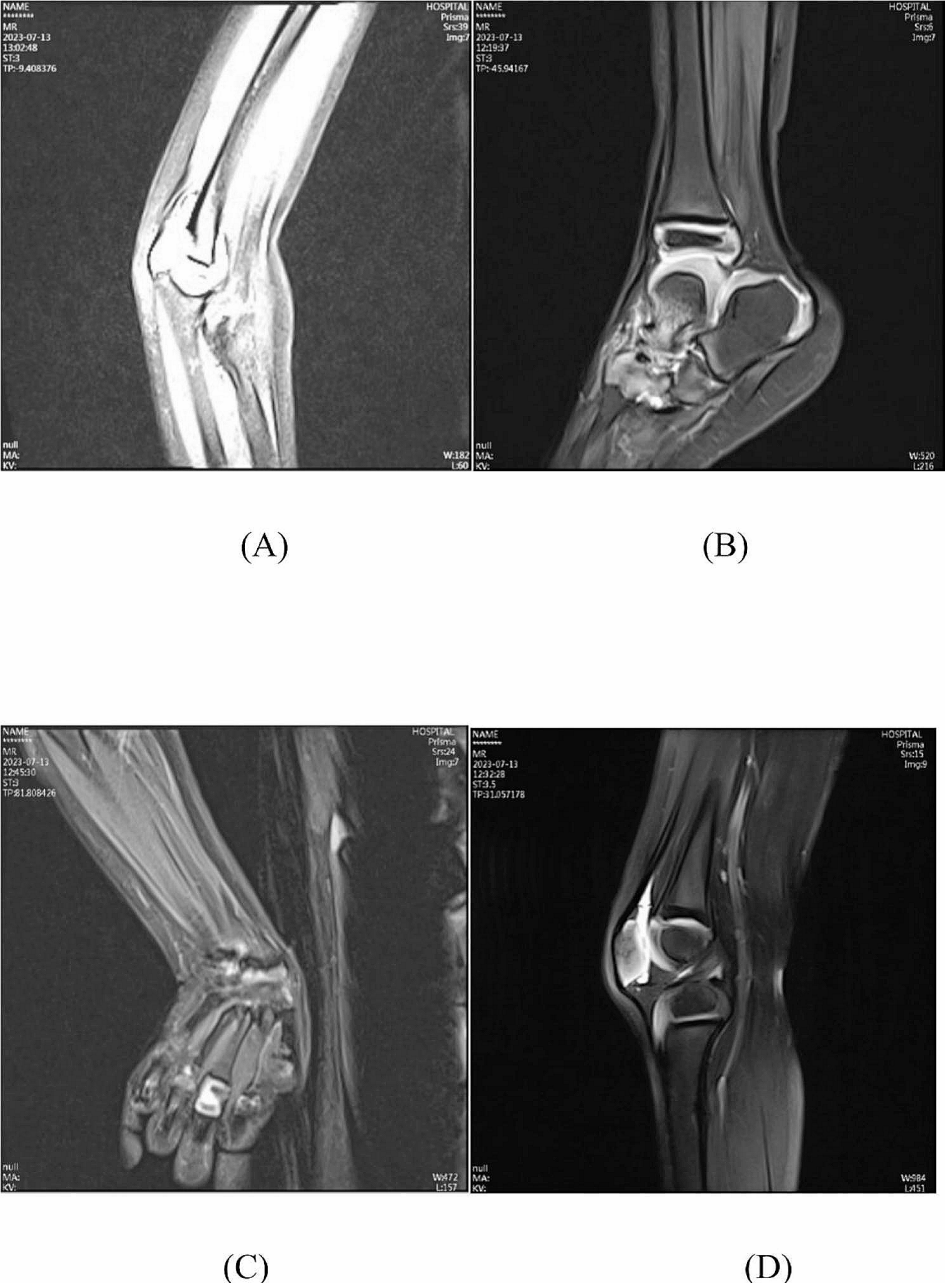
MRI showed decreased articular effusion of the right elbow (**A**) and no deterioration of destruction and bone resorptions of the ankle joint (**B**), metatarsophalangeal joints and interdigital joints (**C**), and left knee joint (**D**) after 1 more year of treatment

The patient was treated with oral methotrexate (7.5 mg/m2 one dose weekly for 2 months and 10mg/m^2^ one dose weekly after that and remaining the dose till his last visit at the age of 4 years) and oral methylprednisolone (equal to oral prednisolone 2 mg/kg/day in the beginning for 3 months and then reduced to 1.25 mg/day gradually over 1 year and remained the dose till his last visit) to alleviate the inflammation of joints. Denosumab, the anti-RANKL (receptor activator nuclear factor κ B ligand) antibody, was administered at a single dose of 20 mg per month for one year and then 60 mg every 2.5 months for 3 times till his last visit. In his last visit, the pain and swelling of joints were partially alleviated and the joint osteolysis had not further deteriorated. The patient regained the ability to walk with limited endurance. The Magnetic resonance imaging (MRI) showed decreased articular effusion of the right elbow and no deterioration of joint destruction and bone resorptions after 1 more year of treatment (Fig. 6).

## Prediction of protein structures and mutant stability

Using NCBI’s Conserved Domain Database [14], MEME (Multiple EM for Motif Elicitation) motif discovery tool [15], the ClustalW program and the ESPript web server [16], the I-tasser web tool [17], the Swiss-model web tool [18], and the I-Mutant server [19] to predict of protein structure and mutation stability.

**Fig. 7:**
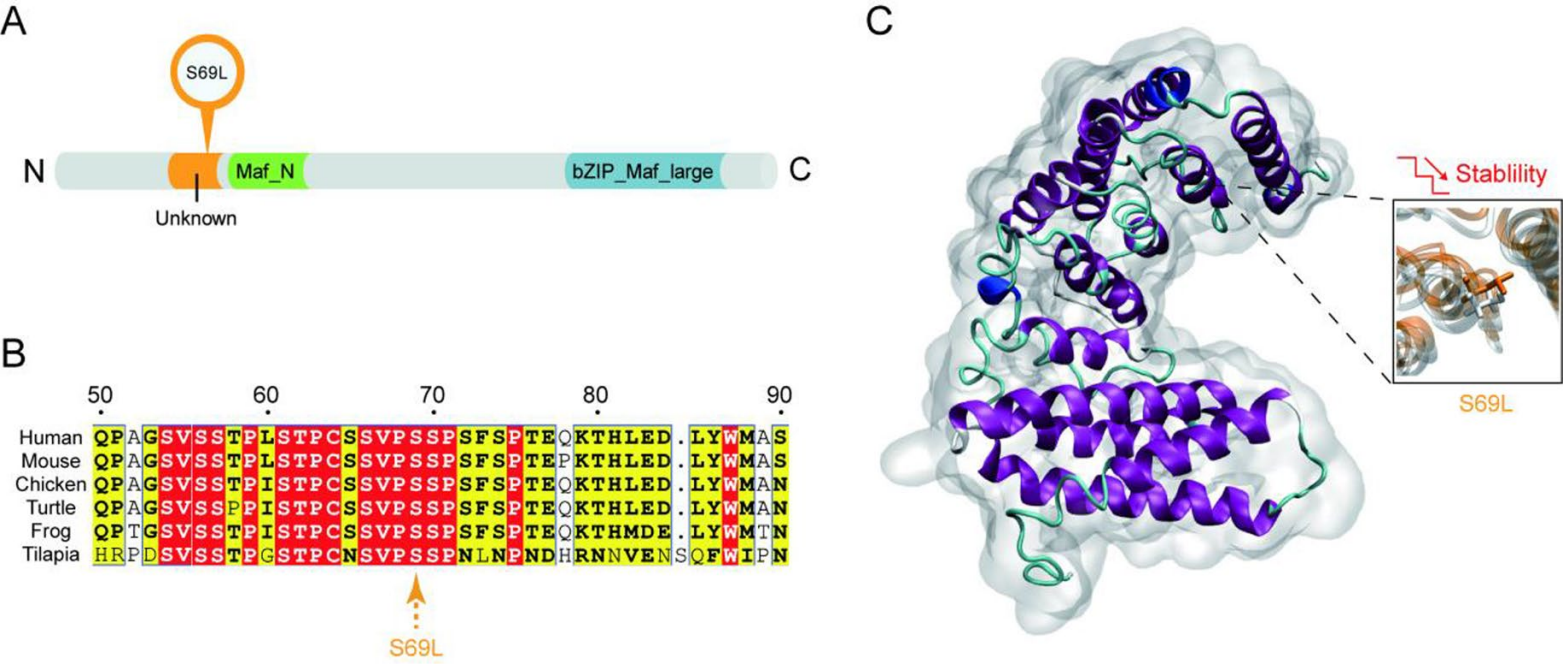
Variation analysis result. One variant of MAFB, S69L, was located inside the unknown domain (**A**). Multiple sequence alignment indicated that the residue S69L was highly conserved among different species (**B**). To visualize the effect of the mutations on the tertiary structure, three-dimensional structural models of ARHGAP35-WT and S69L were predicted and superimposed (**C**)

One variant of MAFB, S69L, was located inside the unknown domain (residues S54-F73, Fig. 7A). Multiple sequence alignment indicated that the residue S69L was highly conserved among different species (Fig. 7B). To visualize the effect of the mutations on the tertiary structure, three-dimensional structural models of ARHGAP35-WT and S69L were predicted and superimposed (Fig. 7C). The overall structure of MAFB adopted a distorted “U” shape, comprisingα-helixes and random coils. S69L was located on a surface-exposed α-helix. The decreased thermo-stability change upon mutation indicated that mutating the neutral charged S69 to hydrophobic Leu might lead to remarkable alteration of local H-bond networks and considerable structural instability under thermal circumstances.

## Discussion and conclusions

The onset age for MCTO varies widely from infancy through adulthood, however, it most commonly manifests during childhood or adolescence [20]. This condition seems irrelative to inflammation with normal CRP and inflammatory cytokines [2]. The pathophysiology behind MCTO involves an abnormal signal pathway between cells within the joint capsule leading to increased production of matrix metalloproteinases (MMPs). These enzymes are responsible for breaking down collagen fibers which form part of cartilage tissue structure. In individuals with MCTO, this process occurs at an accelerated rate causing rapid erosion and thinning out bone structures around joints affected by disease activity. This leads to instability due to weakened ligaments surrounding these areas making them more prone to injury from everyday activities like gripping objects or typing on keyboards etc. Additionally, there may be associated inflammation caused by the release of inflammatory mediators into synovial fluid further exacerbating symptoms experienced during flare-up episodes where patients experience severe pain swelling stiffness limited range of motion difficulty performing daily tasks involving the use of their hands/wrists [20, 21].

Diagnosing MCTO poses significant challenges for healthcare professionals primarily due to its rarity and similarities with various other conditions such as JIA which may lead to misdiagnosis if not carefully evaluated [3, 22, 23]. The initial symptoms of MCTO often include pain, swelling, stiffness, and limited range of motion in the affected joints. These manifestations are typically bilateral but may not occur simultaneously or symmetrically across all patients [22]. The diagnosis of MCTO requires clinical suspicion based on family history combined with imaging studies showing symmetrical erosions involving multiple small joints, especially those located distally on fingers, toes, wrists, ankles, elbows, knees, hips, shoulders, and spine [24]. However, genetic analysis remains the gold standard to confirm diagnosis [2, 25, 26].

Prognosis depends largely upon how early diagnosis is made degree of disability incurred prior to treatment initiation age gender overall health status person living with multicentric carpotarsal osteolysis. Generally speaking, prognosis is good if appropriate interventions are implemented timely manner while outcomes are less favorable for those who delay seeking medical attention until later stages of progression have already set. With proper care and support individuals diagnosed with this condition can lead relatively normal lives despite having lived with daily challenges posed to them their illness.

However, in daily clinical practice, lots of MCTO patients have suffered a very long period of incorrect diagnosis, following inappropriate treatment, resulting a poor outcomes [1, 22]. The delay in diagnosis has been reported to have been a number of decades when there are atypical manifestations, delayed MRI scanning, a negative result of synovial biopsy, a negative targeted genetic detection and some other reasons [1, 22]. Interestingly, the patient with an MCTO family history often get an early-stage diagnosis and better life standard [22]. This indicates that the detection of genetic mutation is essential for recognizing MOCT patients.

Denosumab is an FDA-approved human monoclonal IgG2 kappa immunoglobulin G antibody designed to target RANKL. The RANKL plays a key role in regulating normal skeletal development by controlling the activity of osteoclasts—cells responsible for breaking down old or damaged bones so they can be replaced with new ones during remodeling processes like growth or repair after injury [27, 28]. By binding RANKL molecules at their active sites on cell surfaces throughout the body’s skeleton system, denosumab prevents them from activating receptors found inside cells involved in stimulating production/activity levels necessary for proper functioning; thus reducing overall rates at which existing bones are broken down while also inhibiting formation/growth rate associated with new ones being created [29, 30]. This makes it useful in the treatment of MOCT, less destruction means more preservation even if there isn’t any increase happening elsewhere within the same area(s) [8]. Even though denosumab has potential prospects for the treatment of MCTO, in the condition of rare prevalence of MCTO with low clinical experience of denosumab, more analysis of MCTO cases or cohort on the effectiveness and safety of denosumab in the treatment of MCTO is intensively required [8, 31].

In our case, when the patient visited our hospital, the scan of MRI on the painful joints was undertaken early. Then the diagnosis of MOCT was considered, and the whole exome sequencing proceeded instead of targeted genes related to MOCT such as TREX1 and MMP9, at an early age. The clinical diagnosis was drawn with the detection of the MAFB gene’s genetic defect. After that, the Denosumab which might be the only potential drug for the MAFB mutation was conducted, even though the drug might only release the symptom but can not heal the damaged bone. Fortunately, no deterioration of joint destruction was shown in the last visit of the patient after about 1.5 years of treatment.

In conclusion, the MRI examination and Whole exome sequencing are recommended to be performed in the early stage of the patient who is suspected of MCTO or suffered bone problems with normal inflammation lab examination. Denosumab might be a potential treatment for MCTO.

The limitation of this report is the lack of pathological results for destructive bone. Longer follow-up duration is necessary in future studies.

## Data Availability

Not applicable.

## Abbreviations

MCTO: Multicentric carpo-tarsal osteolysis
MAFB: MAF BZIP Transcription Factor B
MRI: Magnetic resonance imaging
MEME: Multiple EM for Motif Elicitation
JIA: Juvenile Idiopathic Arthritis
BUN: Blood urea nitrogen
CRP: The C-reactive protein
ESR: Erythrocyte Sedimentation Rate
TNF-β: Tumor necrosis factor-β
IL: Interleukin
RANKL: Receptor activator nuclear factor κ B ligand

## References

[1] Drovandi S, Lugani F, Boyer O, La Porta E, Giordano P, Hummel A et al. Multicentric Carpotarsal Osteolysis Syndrome Associated Nephropathy: Novel Variants of MAFB Gene and Literature Review. J Clin Med. 2022;11(15). 10.3390/jcm11154423 PMC9369440 35956038

[2] Tannouri L, Simoni P (2023). Multicentric Carpo-Tarsal Osteolysis. J Belg Soc Radiol.

[3] Wu J, Wang L, Xu Y, Zhang Z, Yan X, An Y (2021). Multicentric Carpo-Tarsal Osteolysis Syndrome Mimicking Juvenile Idiopathic Arthritis: two case reports and review of the literature. Front Pediatr.

[4] Furness L, Riley P, Wright N, Banka S, Eyre S, Jackson A (2022). Monogenic disorders as mimics of juvenile idiopathic arthritis. Pediatr Rheumatol Online J.

[5] Gupta N, Chakraborty S, Chowdhury MR, Puri RD, Jana M, Kumari I (2023). A report of 5 Indian families with multicentric carpotarsal osteolysis syndrome. Eur J Med Genet.

[6] Hamada M, Tsunakawa Y, Jeon H, Yadav MK, Takahashi S (2020). Role of MafB in macrophages. Exp Anim.

[7] Han Y, Shao W, Zhong D, Ma C, Wei X, Ahmed A et al. Zebrafish mafbb mutants display osteoclast over-activation and bone deformity resembling Osteolysis in MCTO patients. Biomolecules. 2021;11(3). 10.3390/biom11030480 PMC8004647 33806930

[8] Regev R, Sochett EB, Elia Y, Laxer RM, Noone D, Whitney-Mahoney K (2021). Multicentric carpotarsal osteolysis syndrome (MCTO) with generalized high bone turnover and high serum RANKL: response to denosumab. Bone Rep.

[9] Chen K, Zamariolli M, Soares MFF, Meloni VA, Melaragno MI (2022). Multicentric Carpotarsal Osteolysis Syndrome in a mother and daughter with a MAFB missense variant and natural history of the Disease. Mol Syndromol.

[10] Kisla Ekinci RM, Ozalp O, Anlas O, Atmis B, Ata A, Balci S (2023). An unusual manifestation in a pediatric patient with MAFB mutation: Sacroiliitis in multicentric carpotarsal osteolysis syndrome. Int J Rheum Dis.

[11] Trinkino B, Ma NS (2023). Treatment of a young child with multicentric carpotarsal osteolysis exhibiting joint inflammation and dysfunctional bone formation. Bone Rep.

[12] Kaimori JY, Mori T, Namba-Hamano T, Morimoto T, Takuwa A, Motooka D (2021). Cyclosporine a Treatment of Proteinuria in a New Case of MAFB-Associated Glomerulopathy without Extrarenal involvement: a Case Report. Nephron.

[13] Miyazaki K, Komatsubara S, Uno K, Fujihara R, Yamamoto T (2019). A CARE-compliant article: a case report of scoliosis complicated with multicentric carpotarsal osteolysis. Med (Baltim).

[14] NCBI. Search for Conserved Domains within a protein or coding nucleotide sequence. Available from: https://www.ncbi.nlm.nih.gov/Structure/cdd/wrpsb.cgi.

[15] Multiple Em for Motif Elicitation. MEME Suite 5.5.4. Available from: https://meme-suite.org/meme/tools/meme.

[16] SBGrid Consortium. ESPript 3.0. Available from: https://espript.ibcp.fr/ESPript/ESPript/.

[17] University of Michigan. I-TASSER Protein Structure & Function Prediction. Available from: https://seq2fun.dcmb.med.umich.edu//I-TASSER/.

[18] Swiss Institute of Bioinformatics. SWISS-MODEL. Available from: https://swissmodel.expasy.org/interactive.

[19] I-Mutant. I-Mutant server. Available from: https://folding.biofold.org/i-mutant/i-mutant2.0.html.

[20] Ma NS, Mumm S, Takahashi S, Levine MA (2023). Multicentric Carpotarsal Osteolysis: a contemporary perspective on the unique skeletal phenotype. Curr Osteoporos Rep.

[21] Zhuang L, Adler S, Aeberli D, Villiger PM, Trueb B (2017). Identification of a MAFB mutation in a patient with multicentric carpotarsal osteolysis. Swiss Med Wkly.

[22] Mehawej C, Courcet JB, Baujat G, Mouy R, Gerard M, Landru I (2013). The identification of MAFB mutations in eight patients with multicentric carpo-tarsal osteolysis supports genetic homogeneity but clinical variability. Am J Med Genet A.

[23] Klein C, Bellity J, Finidori G, Glorion C, Pannier S (2018). Multicentric carpotarsal osteolysis syndrome: long-term follow-up of three patients. Skeletal Radiol.

[24] Zankl A, Duncan EL, Leo PJ, Clark GR, Glazov EA, Addor MC (2012). Multicentric carpotarsal osteolysis is caused by mutations clustering in the amino-terminal transcriptional activation domain of MAFB. Am J Hum Genet.

[25] Li J, Shi L, Lau K, Ma Y, Jia S, Gao X (2020). Identification of a novel mutation in the MAFB gene in a pediatric patient with multicentric carpotarsal osteolysis syndrome using next-generation sequencing. Eur J Med Genet.

[26] Narhi A, Fernandes A, Toiviainen-Salo S, Harris J, McInerney-Leo A, Lazarus S (2021). A family with partially penetrant multicentric carpotarsal osteolysis due to gonadal mosaicism: First reported case. Am J Med Genet A.

[27] Tsourdi E, Langdahl B, Cohen-Solal M, Aubry-Rozier B, Eriksen EF, Guanabens N (2017). Discontinuation of Denosumab therapy for osteoporosis: a systematic review and position statement by ECTS. Bone.

[28] Wan Y, Zeng F, Tan H, Lu Y, Zhang Y, Zhao L (2022). Cost-effectiveness analyses of denosumab for osteoporosis: a systematic review. Osteoporos Int.

[29] Mumm S, Huskey M, Duan S, Wenkert D, Madson KL, Gottesman GS (2014). Multicentric carpotarsal osteolysis syndrome is caused by only a few domain-specific mutations in MAFB, a negative regulator of RANKL-induced osteoclastogenesis. Am J Med Genet A.

[30] Cuevas VD, Anta L, Samaniego R, Orta-Zavalza E, Vladimir de la Rosa J, Baujat G (2017). MAFB determines human macrophage anti-inflammatory polarization: relevance for the pathogenic mechanisms operating in Multicentric Carpotarsal Osteolysis. J Immunol.

[31] Lerman MA, Francavilla M, Waqar-Cowles L, Levine MA (2023). Denosumab Treatment does not halt progression of bone lesions in Multicentric Carpotarsal Osteolysis Syndrome. JBMR Plus.

